# Phytotoxicity and Accumulation of Copper-Based Nanoparticles in *Brassica* under Cadmium Stress

**DOI:** 10.3390/nano12091497

**Published:** 2022-04-28

**Authors:** Shiqi Wang, Yutong Fu, Shunan Zheng, Yingming Xu, Yuebing Sun

**Affiliations:** 1 College of Resources and Environment, Northeast Agricultural University, Harbin 150030, China; wwp875822270@163.com (S.W.); 18845141255@163.com (Y.F.); 2 Key Laboratory of Original Agro-Environmental Pollution Prevention and Control, Agro-Environmental Protection Institute, Ministry of Agriculture and Rural Affairs (MARA), Tianjin 300191, China; ymxu1999@126.com; 3 Tianjin Key Laboratory of Agro-Environment and Agro-Product Safety, Agro-Environmental Protection Institute, Ministry of Agriculture and Rural Affairs (MARA), Tianjin 300191, China; 4 Rural Energy & Environment Agency, Ministry of Agriculture and Rural Affairs (MARA), Beijing 100125, China; zhengshunan1234@163.com

**Keywords:** copper-based nanoparticles, phytotoxicity, bioaccumulation, nutrient element

## Abstract

The widespread use of copper-based nanoparticles expands the possibility that they enter the soil combined with heavy metals, having a toxic effect and posing a threat to the safety of vegetables. In this study, single and combined treatments of 2 mg/L Cd, 20 mg/L Cu NPs and 20 mg/L CuO NPs were added into Hoagland nutrient solution by hydroponics experiments. The experimental results show that copper-based Nanoparticles (NPs) can increase the photosynthetic rate of plants and increase the biomass of *Brassica*. Cu NPs treatment increased the Superoxide Dismutase (SOD), Peroxidase (POD) and catalase (CAT) activities of *Brassica*, and both NPs inhibited ascorbate peroxidase (APX) activity. We observed that Cd + Cu NPs exhibited antagonistic effects on Cd accumulation, inhibiting it by 12.6% in leaf and 38.6% in root, while Cd + CuO NPs increased Cd uptake by 73.1% in leaves and 22.5% in roots of *Brassica*. The Cu content in the shoots was significantly negatively correlated with Cd uptake. The Cd content of each component in plant subcellular is soluble component > cytoplasm > cell wall. Cu NPs + Cd inhibited the uptake of Zn, Ca, Fe, Mg, K and Mn elements, while CuO NPs + Cd promoted the uptake of Mn and Na elements. The results show that copper-based nanoparticles can increase the oxidative damage of plants under cadmium stress and reduce the nutritional value of plants.

## 1. Introduction

Cadmium (Cd) is one of the most biologically toxic heavy metals. Cd pollution in farmland not only reduces soil quality and crop yield, but also threatens the health and well-being of animals and humans through the food chain [1]. The long-term consumption of high levels of Cd can lead to hypercalciuria, renal failure, anemia and even death. Cd can be toxic to organisms even at very low concentrations, and can also be toxic to plants when the total concentration exceeds 8 mg/kg [2]. According to the National Soil Pollution Survey Bulletin, the excess rate of Cd in China reached 7.0, showing a trend of gradually increasing from northwest to southeast and from northeast to southwest [3]. A meta-analysis of heavy metals in Chinese farmland and urban soil showed that Cd was the most commonly polluted heavy metal in Chinese soil, accounting for 33.54% and 44.65% of farmland and urban soil pollution, respectively [4].

Cu-based nanoparticles (NPs) have unique properties such as small volume, large specific surface area, high activation energy and many active sites. They have great application potential in industrial, agricultural and commercial fields. There are data to prove that in 2010, the global production of copper-based NPs was 200 tons per year, and it is increasing year by year [5]. Additionally, some studies are exploring the feasibility of using copper-based nanomaterials as nano-fertilizers [6,7], which increases the risk of their entry into the soil environment due to transportation, application, leakage, etc. Copper-based nanoparticles will be absorbed by plants after entering the soil. At low doses, they will have a stimulating effect on plants, which can promote plant growth and development and improve plant tolerance to adverse environmental stress [8], but high doses cause toxic effects on plants [3]. Metal element/metal oxide nanomaterials can also release metal ions, causing cellular oxidative stress and poisoning plants. Studies have shown that the addition of CuO NPs reduced the seed germination rate and the rhizome length of seedlings, decreased root cell viability, and increased the generation of plant reactive oxygen species (ROS) and lipid peroxidation [9,10]. Cu NPs can alter the activity of antioxidant enzymes in plants and activate the antioxidant enzyme defense mechanism against ROS [11]. Copper-based NPs can inhibit the accumulation of nutrient elements, thereby affecting human nutrient intake [12]. Copper-based NPs also affect plant genes; studies have shown that when plants are exposed to different concentrations of CuO NPs, the expressions of the CuZn-superoxide dismutase (CuZnSOD) gene, CAT gene and APX gene in roots are increased to varying degrees [10]. Depending on the plant species, the applied concentration of NPs and the particle size of NPs, different toxic effects will be exhibited. 

In addition to causing nanotoxicity, nanoparticles can also adsorb other pollutants, and when they enter the environment, they can act synergistically or antagonistically with heavy metals present in the environment. At present, some studies have investigated the plant performance of other nanomaterials under the combined stress of heavy metal cadmium, but different types of NPs and heavy metals, application ratios, plant species and culture conditions will lead to different toxic effects. Cd^2+^ and TiO_2_ NPs exhibited different combined toxicity patterns against *Scenedesmus obliquus* at different combined exposure concentrations. Antagonistic effects were exhibited at low doses, and partial additive and synergistic combined toxicity occurred when the proportion of TiO_2_ NPs was increased [13]. ZnO NPs at a concentration of 25 mg/L promoted the growth of *Leucaena Leucocephala* seedlings under cadmium and lead stress, increased the activities of SOD, CAT and other antioxidant enzymes, and significantly decreased the malonaldehyde (MDA) content [14]. After wheat seeds soaked in Fe NPs and ZnO NPs solution were sown in Cd-contaminated soil, the dry weight of wheat was positively correlated with the amount of NP added, which significantly reduced the Cd content of each part of the plant [15]. To date, most studies have focused on the negative effects of copper-based nanomaterials or cadmium alone on plants, and it is crucial to explore the physiological performance and toxicity mechanisms of plants under the combined stress of the two. 

*Brassica* (*Brassica campestris* L. ssp. *chinensis* Makino var. *communis* Tsen et Lee) is widely grown in the north and south of China and has strong enrichment for heavy metal Cd. In this experiment, parameters such as photosynthetic rate, biomass, antioxidant enzyme activity, plant absorption of heavy metals, nutrient element content and plant subcellular element content were determined, in order to explore the combined toxicity of copper-based nanoparticles and Cd in plants. This provided a reference for the in-depth study of the toxicity mechanism of copper-based nanoparticles combined with heavy metals. 

## 2. Materials and Methods

### 2.1. Cu and CuO NPs Characterization

Nanomaterials were purchased from Zhejiang Ailu Chemical Technology Co., Ltd. (Li Shui City, China). The characterization revealed that the purity of Cu NPs exceeded 99.9%, with a size of 10−30 nm and specific surface area of 6.99 m^2^·g^−1^. CuO NPs purity exceeded 99.5%, particle size was 40 nm and specific surface area was 2.84 m^2^·g^−1^.

### 2.2. Hydroponic Experiment Design and Exposure Conditions

Seeds of *Brassica* were purchased from Cangzhou Heshuo Agricultural Technology Co., Ltd. (Cangzhou, China). The seeds were washed in deionized water, and the growing medium was vermiculite. After the seedlings were taken out and the roots carefully washed, the *Brassica* seedlings were transferred to hydroponic boxes and incubated with Hoagland nutrient solution for 20 days. The ratio of Hoagland nutrient solution is shown in Table S1. For nanoparticle exposure, according to the effective concentration of previous hydroponic experiments [11,16,17,18], we set the experimental NPs concentration as 20 mg/L. Five treatments were designed, including Cd (2 mg/L Cd), Cu NPs (20 mg/L Cu NPs), CuO NPs (20 mg/L CuO NPs), Cd + Cu NPs (2 mg/L Cd + 20 mg/L Cu NPs), and Cd + CuO NPs (2 mg/L Cd + 20 mg/L CuO NPs). Each treatment was repeated three times. Each hydroponic box was equipped with an aerator to provide oxygen to the roots while keeping the NPs in suspension. After culturing for 7 days, all the *Brassica* plants were collected to measure various indicators.

### 2.3. Determination of Physiological Indicators

The photosynthetic rate (Pn) of *Brassica* was measured by a portable photosynthesis measurement system at 10:00 a.m. every day. After 7 days, plants were separated into underground parts and aboveground parts and soaked in 10 mmol·L^−1^ Na_2_-EDTA solution for 40 s, to remove metal ions adhering to the root surface. After rinsing with ultrapure water, each part of the fresh sample was weighed with an analytical balance. The fresh samples were refrigerated at −80 °C for later use. The *Brassica* tissues were oven-dried for 7 days at 80 °C and weighed to record the biomass. The oven-dried tissues were ground to powder for subsequent use.

### 2.4. Determination of Cu, Cd and Other Nutrient Elements

Approximately 0.25 g of the sample was soaked and digested with 8 mL of concentrated HNO_3_, and the contents of copper, cadmium and nutrients in *Brassica* were determined by inductively coupled plasma mass spectrometer (ICP-MS, Waltham, MA, USA).

### 2.5. Determination of Subcellular Cu, Cd and Nutrient Elements

The experimental method refers to Li et al. [19]. A total of 3 g of fresh samples was centrifuged at 2000 rpm at 4 °C for 5 min in the pre-cooled extraction solution (50 mM Tris–HCl, 250 mM sucrose, and 1.0 mM DTE (C_4_H_10_O_2_S_2_), pH 7.5). The precipitate was obtained and defined as the ‘cell wall fraction’. The filtrate was transferred to a special tube for a refrigerated centrifuge and centrifuged at 11,900 rpm at 4 °C for 45 min. The deposit was referred to as the ‘organelle fraction’, and the supernatant solution as the ‘soluble fraction’. After the drying and digestion of each component, ICP-MS was used for determination.

### 2.6. Determination of Antioxidant Enzyme Activity

The enzyme activity of *Brassica* was measured in leaves and root. Peroxidase (POD) activity was determined by the guaiacol method, CAT activity was determined by the potassium permanganate titration method, and superoxide dismutase (SOD) activity was determined by the nitrogen blue tetrazolium photoreduction method [20].

Ascorbate peroxidase (APX) activity was determined as follows: 0.1 g of plant tissue was homogenized in an ice bath and centrifuged at 4 °C for 20 min. To the supernatant was added K_2_HPO_4_-KH_2_PO_4_ buffer, 0.3 mmol·L^−1^ ascorbic acid (AsA), 0.1 mmol·L^−1^ EDTA-2NA and 0.06 mmol·L^−1^ H_2_O_2_. After rapid mixing, the absorbance was measured at 290 nm for 10 and 130 s [21]. The unit of antioxidant enzyme activity is U‧g^−1^.

### 2.7. Statistical Analysis

The results are presented as means ± standard errors of 3 replicates. One-way analysis of variance (one-way ANOVA) was used to determine statistical differences between treatments, followed by an LSD test performed by IBM SPSS Statistics 26. “*p* < 0.05” was used for statistical significance.

## 3. Results

### 3.1. Photosynthesis and Plant Growth

The photosynthetic rate was measured from the first to the seventh day of hydroponics of *Brassica* (Table 1). The treatments showed a more obvious change on days 5–7 as the experimental time increased. Consistent with previous research [22,23], compared with CuO NPs treatment, heavy metal Cd significantly inhibited the photosynthetic rate of *Brassica* by 10.02–12.64%. In the measurement of photosynthetic rate in each group within seven days, the photosynthetic rate of CuO NPs treatment was the highest, and the photosynthetic rate of other treatments showed different degrees of decline. Compared with CuO NPs treatment, the photosynthetic rate of the Cu NPs group decreased by 1.07–4.47%. The copper-based nanoparticles alleviated the stress of Cd on the photosynthesis of *Brassica* to a certain extent. Compared with the Cd treatment group, the photosynthetic rate of the Cu NPs + Cd group and the CuO NPs + Cd group both increased, and the results were similar to those of the treatment without heavy metals. In contrast, CuO NPs showed a more significant photosynthesis promotion effect, being increased by 10.2–19.6%. Although the increase in photosynthetic rate was also observed in the Cu NPs group, most changes were not significant.

**Figure 1:**
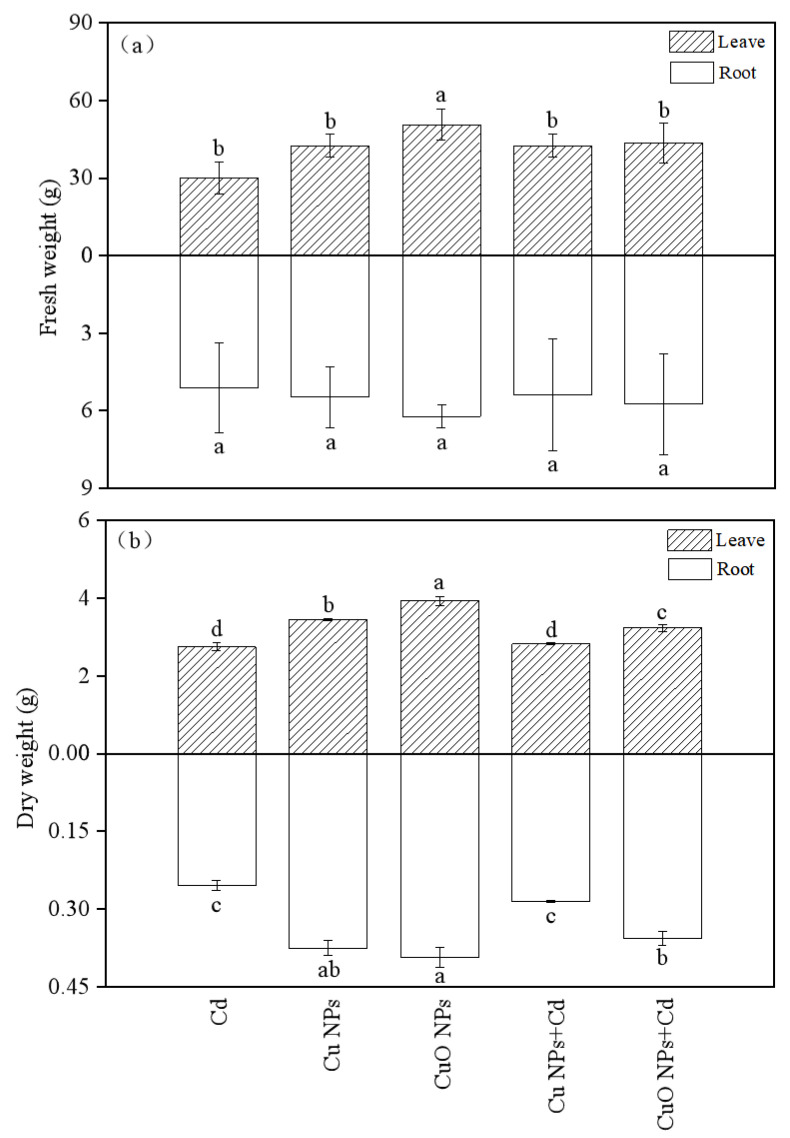
Effect of Cu NPs, CuO NPs and Cd on fresh weight (**a**) and dry weight (**b**) of *Brassica*. The means are averaged from three replicates, and the error bars correspond to the standard deviations of the three values. Different letters above each column indicate a significant difference among treatments in the same group (*p* < 0.05).

Figure 1 showed the measurement results of the fresh and dry weights of the leaves and roots of *Brassica*, root fresh weight was not significantly affected by any of the treatments. Cd had a certain inhibitory effect on the biomass of *Brassica*. The fresh and dry weights of *Brassica* under the CuO NPs treatment were the largest among the five treatments (50.65 g and 6.22 g in the shoots, and 3.93 g and 0.37 g in the roots, respectively). Cu NPs and CuO NPs promoted the fresh weight increase of *Brassica* under Cd stress by 41.0% and 44.4%, respectively. Compared with the Cd group, the dry weight of *Brassica* in the Cd + Cu NPs treatment showed a slight increase (2.5% above ground and 3.2% below ground), but these data were not statistically significant. A significant increase in dry weight was observed in the Cd + CuO NPs group (17.4% above ground and 39.8% below ground). This indicates that the two copper-based nanoparticles can alleviate the inhibitory effect of Cd on the biomass of *Brassica* to a certain extent, and the effect of CuO NPs is more obvious.

### 3.2. Antioxidase Activity

**Figure 2:**
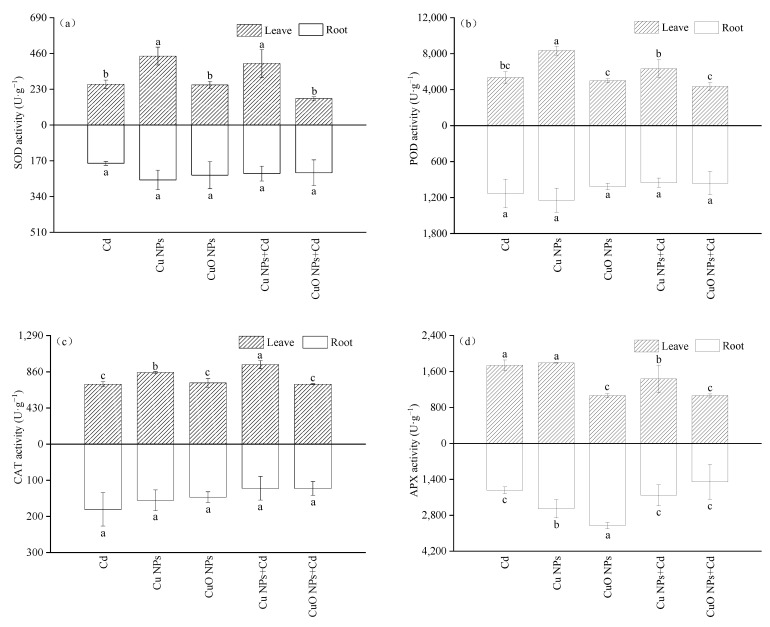
Effect of Cu NPs, CuO NPs and Cd on SOD activity (**a**), POD activity (**b**), CAT activity (**c**) and APX activity (**d**) of *Brassica*. The means are averaged from three replicates, and the error bars correspond to the standard deviations of the three values. Different letters above each column indicate a significant difference among treatments in the same group (*p* < 0.05).

In this experiment, the activities of SOD, POD and CAT in the underground parts of each treatment did not change significantly (Figure 2). The activities of four antioxidant enzymes in the leaves of Brassica treated with CuO NPs were significantly lower than those of Cu NPs (SOD, POD and CAT activities decreased by 41.8%, 40.0% and 14.58%, respectively). The SOD, POD, and CAT activities of the three treatments contaminated with Cd were Cd + Cu NPs > Cd > Cd + CuO NPs. Compared with the Cd treatment group, Cu NPs promoted the SOD, POD and CAT activities of *Brassica* under Cd stress by 51.3%, 18.2% and 32.65%, respectively. However, the increase in POD activity in the Cu NPs + Cd group was not significant. In contrast to Cu NPs, although CuO NPs did not significantly change the SOD, POD, and CAT activities of Cd-treated *Brassica*, a weak decrease in enzymatic activity was observed. The above-ground and below-ground activities of APX under the treatments of Cu NPs and CuO NPs showed opposite effects. The activity of the APX group in the Cu NPs leaves was much higher (1.68 times) than that in the CuO NPs group, while the APX activity in the roots decreased by 20.6% compared with the CuO NPs treatment. Under cadmium stress, NPs did not significantly change the APX activity in roots. It was observed that compared with Cd treatment, the APX activity of the Cu NPs + Cd and CuO NPs + Cd groups was significantly inhibited in the shoots, being reduced by 17.26% and 38.49%, respectively.

### 3.3. Uptake of Cu and Cd

**Figure 3:**
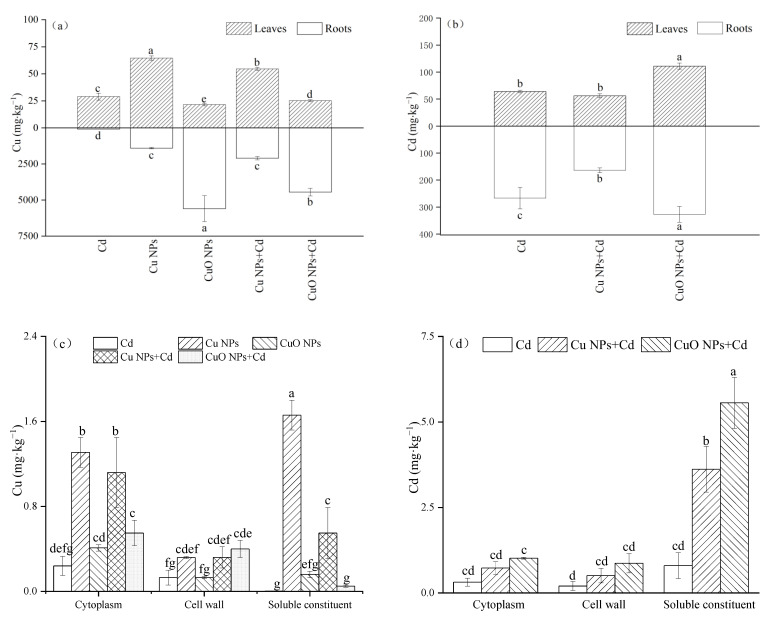
The effects of Cu NPs, CuO NPs and Cd on Cu (**a**), Cd (**b**), subcellular Cu (**c**) and subcellular Cd (**d**) content. The mean is the mean of three replicates and the error bars correspond to the standard deviation of the three values. Different letters above each column indicate significant differences among treatments in the same group (*p* < 0.05).

The Cu and Cd contents in *Brassica* were determined by the above method (Figure 3). Comparing the two types of nanoparticles, the Cu NPs-treated aboveground Cu accumulation was 2.99 times that of the CuO NPs-treated group, but the underground Cu content (1402.07 mg‧kg^−1^) was much lower than that of the CuO NPs group (5594.39 mg‧kg^−1^). For the aboveground Cu content of the Cd + Cu NPs and Cd + CuO NPs groups, we observed two opposite influence trends. Compared with Cd treatment, Cu NPs increased by 88.4% while CuO NPs decreased by 15.4%, both trends showing significant changes. Different from the above-ground part, both Cu-containing nanoparticles significantly promoted the accumulation of Cu content in the underground part of *Brassica*, which was 25.58 times and 54.10 times that of the Cd treatment, respectively. The total copper content in *Brassica* in the CuO NPs + Cd group was much higher than that in the Cu NPs + Cd group, but it was mostly accumulated in the roots of *Brassica*, and only a small amount was transported to the shoots.

By comparing the Cd content of *Brassica* after adding two kinds of copper-based nanoparticles, it was found that compared with Cd treatment, Cu NPs showed an inhibitory effect on the absorption of heavy metal Cd in *Brassica*. Cu NPs + Cd decreased by 12.6% and 38.6% in leaves and roots, respectively, but there was no significant difference in the changes in leaves. On the contrary, the addition of CuO NPs increased the accumulation of Cd in the plants. Compared with the Cd group, the Cd content in the leaves and roots of *Brassica* increased significantly, increasing by 73.1% and 22.5%, respectively.

The subcellular Cu and Cd contents were determined using fresh *Brassica* leaf samples from each treatment, and the subcellular Cu content in the shoots is shown in Figure 3c. The subcellular Cu content of *Brassica* after Cu NPs treatment was higher than that of CuO NPs treatment. This is consistent with the results for the total Cu content in the shoots. Under Cd pollution, the copper-containing nanoparticles all caused the Cu content in each component in the subcellular to increase significantly. Compared with the Cd group, the proportion of Cu content in the soluble fraction of the Cu NPs + Cd treatment increased significantly, while the proportion of Cu content in the cell wall in the CuO NPs + Cd group also increased significantly. The Cd content in each subcellular component also showed an upward trend: the Cd content in Cu NPs + Cd and CuO NPs + Cd organelles increased by 130.8% and 221.9%, in the cell wall by 151.4% and 330.3%, and in the soluble fraction by 353.7% and 597.6%, respectively. The subcellular Cd content of *Brassica* mainly accumulated in the soluble fraction of cells.

### 3.4. Nutrient Element Content

The effects of different treatments on the nutrient content of *Brassica* are shown in Table 2. Compared with the Cu NPs treatment, the contents of nutrient elements in the CuO NPs group were increased in the underground part, while the contents of other elements in the aboveground parts were not statistically significant, except for Mn and Na elements, which decreased significantly. Compared with the Cd group, the contents of nutrient elements in roots measured in Cu NPs + Cd and CuO NPs + Cd treatments all increased to varying degrees. The change in Fe and Ca elements was consistent, and the content of aboveground elements decreased significantly. Fe content in leaves decreased significantly by 46.6% and 43.1%, and Ca content decreased by 22.86% and 17.48%. Cu NPs also significantly inhibited the absorption of Zn, Mg, and K elements in the leaves of *Brassica* under Cd treatment. The contents of Ca in the upper part of the ground decreased after the addition of CuO NPs. Cu NPs reduced the uptake of Mn elements in leaves, whereas CuO NPs showed a promotion effect on the uptake of Mn and Na elements (the contents of Mn and Na elements in leaves were increased by 24.3% and 28.3%, respectively).

**Figure 4:**
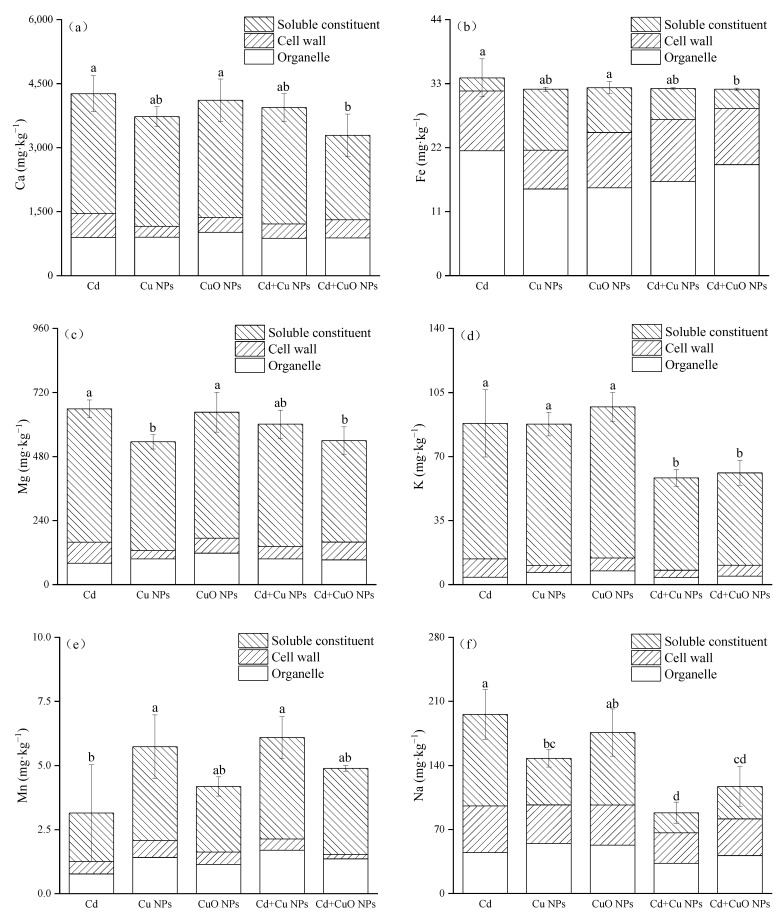
Effects of Cu NPs, CuO NPs and Cd on the subcellular contents of Ca (**a**), Fe (**b**), Mg (**c**), K (**d**), Mn (**e**) and Na (**f**). The mean is the mean of three replicates and the error bars correspond to the standard deviation of the three values. Different letters above each column indicate significant differences between treatments in the same group (*p* < 0.05).

Figure 4 is the determination result of the change in nutrient elements in the subcellular components of *Brassica*. Comparing the two NPs, only the Mg content of CuO NPs was significantly increased, by 20.7% compared with Cu NPs. The subcellular contents of other nutrients were not statistically significant. Except for Mn, the addition of NPs all decreased the content of nutrient elements in the subcellular components of *Brassica* under cadmium stress. Compared with the Cd group, Cu NPs + Cd and CuO NPs + Cd increased the content of Mn element in the subcellular plant components by 93.66% and 55.43%, respectively, but the changes in CuO NPs treatment showed no significant effect. The same decreasing trend was observed for the subcellular contents of the three nutrient elements Ca, Fe, and Mg by NPs. CuO NPs significantly reduced the subcellular content of these three elements by 22.84%, 5.76%, and 18.10%, but Cu NPs did not have significant effects. Compared with Cd treatment, copper-based nanoparticles significantly inhibited the absorption of subcellular K and Na elements, which were reduced by 33.83% and 54.91% in the Cu NPs + Cd group, and 30.70% and 40.23% in the CuO NPs + Cd group.

## 4. Discussion

Cadmium toxicity to plants was observed at the whole plant as well as at the cellular and molecular levels, including energy transfer, photosynthesis, nutritional dysregulation and protein synthesis. Cd can significantly inhibit the photosynthetic rate and growth of plants, and photosynthesis is highly sensitive to Cd and other heavy metal ions [24]. In the present study, copper-based nanoparticles promote the photosynthesis rate of *Brassica* under Cd stress (Table 1). Metal nanoparticles can promote photosynthesis in plants and increase the content of chlorophyll and carotenoids in plants [25]. Govorov et al. believe that metal NPs can improve the efficiency of chemical energy generation in photosynthetic systems [26]. Some studies have shown that copper-based nanoparticles can play a key role in photosynthesis by enhancing chloroplast photosynthetic activity by modulating fluorescence emission, the electron transport chain (ETC), carbon assimilation pathways and photophosphorylation [27]. Plant photosynthesis is very sensitive to Cu [28,29]. Cu plays important roles in mitochondrial respiration, the electron transport chain, photosynthesis, cell wall metabolism and lignin synthesis [30]. Lower concentrations of Cu can promote plant photosynthesis to a certain extent, and the effect is manifested in plant net photosynthetic rate, transpiration rate, relative chlorophyll content and PSII photochemical effect [24]. In the physiological and biochemical study of combined Cu and Cd pollution on *Cinnamomum Camphora*, low-dose Cu promoted the photosynthesis of plants under cadmium stress, and alleviated the damage caused by cadmium to plant photosynthesis to a certain extent [31]. On one hand, the promoting effect of copper-based nanoparticles on *Brassica* biomass is due to the positive effect of photosynthesis on plant growth. On the other hand, lower doses of nanomaterials have a certain stimulatory effect on plants, which can promote plant growth and development and improve plant tolerance to adverse environmental stresses. When the concentration of NPs selected in the experiment is low, the released copper ions can be used as trace elements to stimulate plant growth and increase the biomass of plants [32,33]. Studies have shown that adding a small amount of copper-based nanoparticles can promote plant growth, 10 mg/L CuO NPs increased the root biomass of conventional cotton and transgenic cotton [34]. Low concentrations of nano-copper dioxide particles also had a positive effect on the growth of corn seedlings [8].

Heavy metal stress can negatively affect plant enzyme activity and may cause oxidative damage to cell membranes [35]. Copper-based nanoparticles are phytotoxic by producing excess reactive oxygen species (ROS) or releasing high concentrations of ions [36]. The excessive production of ROS can lead to lipid peroxidation, protein structure destruction, apoptosis and DNA damage [37]. To alleviate the stress caused by ROS, cells have enzymatic mechanisms to eliminate or reduce their damaging effects. Antioxidant enzymes such as catalase (CAT), peroxidase (POD), ascorbate peroxidase (APX) and superoxide dismutase (SOD) play an important role in scavenging ROS to prevent oxidative damage [38]. Antioxidative enzymes are mediators of oxidative damage that help plant biomolecules defend against ROS attack [39]. In plants, CAT is used to remove most of the H_2_O_2_, POD can sequester the remaining H_2_O_2_ [40], and SOD catalyzes the conversion of superoxide to oxygen and hydrogen peroxide. In the present study, the CAT, SOD, and POD activities of *Brassica* treated with Cu NPs + Cd were improved (Figure 1), which was similar to the experimental results of Karimi [41] and Kim [42]. Kim et al. found that the enzyme activities of CAT, SOD and POD in cucumbers grown under hydroponic conditions treated with NPs also showed an upward trend. Cu NPs induce oxidative stress in *Brassica*, and promote and stimulate ROS production, and plants enhance the activity of antioxidant enzymes as a defense system in order to resist ROS stress. However, the activity of antioxidant enzymes was decreased by CuO NPs, indicating that CuO NPs produced more ROS and more strongly inhibited the activities of antioxidant enzymes than Cu NPs. Due to their oxidation state, CuO NPs are more toxic than Cu NPs. The CuO NPs destroyed the plant structure and reduced the amounts of nutrients absorbed by the plant. Nutrients cannot support the normal metabolic activities of plants, and cannot synthesize proteins smoothly, thus inhibiting the synthesis of CAT, SOD and POD. APX has the strongest affinity with H_2_O_2_, which can quickly remove excess H_2_O_2_ produced in cells and protect cells from reactive oxygen species poisoning [43]. In this experiment, copper-based nanoparticles both decreased the APX activity in leaves. This result is different from that of increased APX activity in most studies of NP-stressed plants. In an exposure study of Cu NPs and CuO NPs to lettuce (*Lactuca sativa*), the concentration of 10 mg/L also reduced the APX activity of lettuce [44]. We speculate that the result may be related to the applied NPs concentration, but the mechanism is currently unclear and further research is needed.

Consistent with our expected results, the application of copper-based nanoparticles greatly enhanced the copper content in *Brassica*. Copper-based nanometals and nanometal oxides release metal ions which have toxic effects on plants (such as Cu^2+^). Changes in plant root exudates and culture medium pH can both increase the concentration of soluble metal ions in the rhizosphere solution, so the uptake of metals by plants will also increase [45]. Because of the characteristics of small NPs particles and the large specific surface area, they will adhere to the surface of the root of *Brassica* in large quantities in the culture medium [46]. They can enter plant cells by binding to water channels, carrier proteins, or ion channels. NPs can also increase the permeability of plant cell walls, generate new pores and enter plant cells by endocytosis or by combining with organic chemicals in the environmental medium [47]. The total copper content in Brassica was higher in CuO NPs treatment than in Cu NPs treatment, which is also consistent with the results of Kadri et al. They believed that CuO NPs would release more copper ions than Cu NPs [48]. We observed more Cu accumulated in the roots of *Brassica* than in the leaves. This is due to the plant’s repulsion mechanism that can allow metals to accumulate in the roots and prevents their transport to the shoots to maintain the homeostasis of mineral elements in tissues and organs. Since roots are directly exposed to heavy metal pollutants under hydroponic conditions, most of the cadmium uptake by plants is also concentrated in the roots. We believe that the cadmium content in *Brassica* is related to the copper content transported to the shoot. Usually, different metals may share the same transporter on the cell membrane, and plants do not clearly distinguish between important micronutrients and non-essential metals [49]. Studies have shown that plants absorb micronutrients faster than non-essential heavy metals [50]. Since Cd and Cu are metal elements with the same electronic valence, there are competing sites, so Cu has a certain inhibitory effect on the absorption and transfer of Cd elements, which will reduce the Cd content in the shoots of plants. Consistent with the research results of Cvjetko et al., Cu can effectively inhibit the absorption of Cd when copper and cadmium are combined to stress plants [51]. It was observed that the content of Cd absorbed by the roots of *Brassica* in the treatment group of CuO NPs increased. The hydroxyl, carboxyl, amino, sulfhydryl and aldehyde groups of proteins and polysaccharides in plant cell walls can combine with metal cations through complexation, precipitation and ion exchange, limiting the transport of metal cations through the cell membrane [19]. NPs exposure induces ROS generation or other effects that may alter the cell wall structure, such as reducing cell wall thickness, triggering cell wall loosening, or changing cell wall pore size, which may also lead to an upward trend in Cu and Cd content in plants [52,53]. On the other hand, the higher the total metal content in plants, the more severe the oxidative damage to biomolecules. When the content of the two metal elements in the stressed plants is large, the absorption of the two elements by the plants will show a certain additive effect. For example, in the combined lead and cadmium treatment of soybean, as the lead addition increased, the cadmium content in the roots also increased [54]. Additionally, studies have shown that Cu at low concentrations can alleviate Cd-induced damage, while the combined stress of higher concentrations of Cu and Cd exceeds the potential of plants to resist heavy metal stress, and Cd and Cu may interact to increase Cd uptake by plants [55]. Cu NPs + Cd and CuO NPs + Cd used to treat the subcellular elements resulted in increased Cu and Cd content compared with Cd treatment, which verifies the above conjecture that NPs damage the cell membrane and increase the content of Cu and Cd in plants. The cell wall is considered the first barrier to protect protoplasts from toxicity [56]. The Cu content in the subcellular Cu NPs-treated group was much higher than that in the CuO NPs-treated group, which may be because the particle size of Cu NPs we chose is smaller than that of CuO NPs. In the case of agglomeration, nanoparticles with diameters over 20 nm are barely able to penetrate the cell wall [54,57]. The order of Cd content in each component is soluble component > organelle > cell wall. The contents of Cu and Cd in the subcellular soluble fraction of *Brassica* leaves were high, indicating that the soluble fraction in leaf cells was the main part of Cd enrichment. The soluble components of plants are composed of cytoplasm and vacuoles. Cytoplasm is the main location for cell metabolism, and the function of vacuoles is mainly to participate in cellular water metabolism. Various proteins, organic acids, organic bases and other substances contained in vacuoles can interact with Cd combinations. Following the ingestion of metals, plants limit the excess accumulation of metal elements in their roots or store them in tissues or organs that are less sensitive to their toxicity, which may include the cytoplasm and chloroplasts. It is also one of the important mechanisms by which plants resist heavy metal poisoning and participate in heavy metal detoxification [58,59].

The absorption and content of nutrient elements in roots showed an upward trend (Table 2). The effect may be due to the interaction of heavy metals with metal transporters, resulting in altered plant gene expression related to nutrient uptake and changes in plasma membrane permeability when plants are exposed to cadmium and excess copper stress [60,61]. The content of Fe in the roots increased and the content in the shoots decreased significantly. This may be due to the fact that in copper-stressed plants, iron may be complexed in the form of ferritin (iron-binding protein), which is overproduced to protect cells from oxidative damage [62]. However, Fe was more active than Cu, formed insoluble hydroxides, and remained in the roots [11]. NPs reduced the content of most nutrient elements in the leaves of *Brassica*. On the one hand, the surface effect of NPs may lead to the absorption of a large amount of other useful mineral elements on the surface of NPs, thereby reducing the bioavailability of mineral elements by plants [46]. On the other hand, the Cu ions released by NPs will also have negative effects on the nutrient absorption of *Brassica*. Consistent with previous research results, when the Cu concentration caused stress to plants, the contents of Fe, Ca, K, and Mg in the underground parts of plants were significantly higher than those of the control. On the contrary, the contents of Zn, Ca, K, and Mg in the upper part of the ground all decreased after the addition of NPs [63]. Nano-CuO significantly reduced the absorption of manganese, zinc, iron, magnesium, molybdenum and boron by cotton [34]. Cu NPs and CuO NPs also reduced the absorption of manganese, calcium, phosphorus and magnesium by lettuce in Trujillo Reyes et al. [45]. Cu NPs also reduced the uptake of Mn elements in leaves, whereas CuO NPs showed a promotion effect on the uptake of Mn and Na elements (the contents of Mn and Na elements in leaves were increased by 24.3% and 28.3%, respectively). The addition of NPs changed the ability of *Brassica* to absorb and transport certain nutrients and thus their nutritional value. From the experimental results, the negative impact of Cd combined with copper-based NPs on the nutrient absorption of *Brassica* is far more than that of NPs. The results of subcellular nutrient determination were basically consistent with the results in Table 2. Compared with Cd treatment, the content of most nutrient elements in the subcellular components also showed a downward trend after the addition of NPs. The rise of the subcellular Mn element may be due to it being an important part of the Mn-superoxide dismutase (SOD) enzyme [64]. Plants need to increase absorption to synthesize antioxidant enzyme in order to protect plants from active oxygen damage. In general, NPS interferes with the absorption of the upper nutrient elements of the *Brassica*, which reduces the nutritional value of *Brassica*.

**Figure 5:**
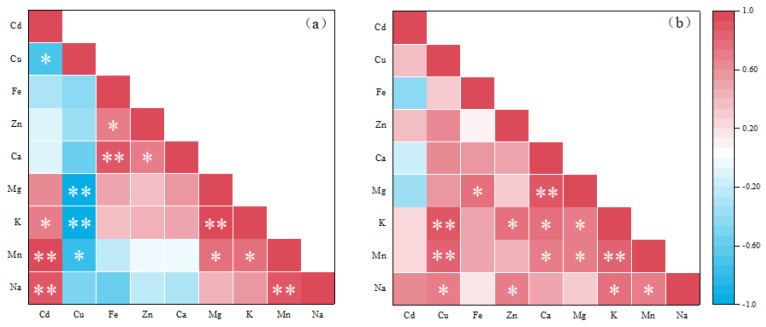
Pearson correlation between antioxidant enzyme activity, heavy metals and mineral elements in *Brassica* leaves (**a**) and roots (**b**). (significant correlation between * and ** at *p* ≤ 0.05 and *p* ≤ 0.01, respectively).

The Pearson correlation results between the elements absorbed by the leaves of *Brassica* are shown in Figure 5a. The absorption of Cd and Cu in the shoots showed a significant negative correlation (ρ = −0.685), which verifies that the two elements are increasing in the plants. When these two elements are transported in the shoots, there is a competitive relationship. When the concentration is low, the effects of Cd and Cu on plants are antagonistic. However, in the experiment, Cd was significantly positively correlated with K (ρ = 0.733), and extremely significantly positively correlated with Mn (ρ = 0.981) and Na (ρ = 0.931). Previous studies have shown that in some plants, both Cd and K are positively correlated. There may be a certain synergistic effect between the elements, and Na is also not negatively affected by Cd treatment [65]. In this study, Cu showed a negative correlation with the absorption of several elements measured, was moderately correlated with Mn (ρ = −0.768), and showed a very high correlation with K and Mg (ρ = −0.954 and ρ = −0.974, respectively). This may be due to the higher copper content altering membrane permeability and impairing plant nutrient uptake. Among the nutrients absorbed by the leaves of *Brassica*, Zn and Fe (ρ = 0.711), Zn and Ca (ρ = 0.730), Mn and Mg (ρ = 0.738), and Mn and K (ρ = 0. 794) showed a significant positive correlation. The correlations between Fe and Ca (ρ = 0.872), Mg and K (ρ = 0.936), and Mn and Na (ρ = 0. 906) were extremely significant and positive.

Figure 5b shows the root element correlation. It is observed that only Cd has a negative correlation with Mg, Ca and Fe, but it is not significant. All other underground elements are positively correlated, among which Cu is extremely significantly positively correlated with Mn and K (ρ = 0.838 and ρ = 0.895, respectively), Ca and Mg (ρ = 0.886), and K and Mn (ρ = 0.812). This may be due to the fact that Cu can increase the permeability of the membrane [66]. NPs can also cause damage to the cells in the root of the plant, which will increase the content of elements entering the *Brassica* [17].

## 5. Conclusions

Copper-based nanoparticles are currently widely used, and may combine with the existing heavy metal pollution in soil to have a combined effect on plants, causing negative effects on crops and humans. In this study, Cu and CuO NPs showed promoting effects on both the photosynthetic rate and biomass of Cd-stressed plants. The changes in antioxidant enzymes indicated that NPs enhanced the oxidative damage of *Brassica* under Cd treatment, and the oxidative damage caused by CuO NPs was stronger than that of Cu NPs. Both applications of copper-containing nanoparticles increased the Cu content in plants. Cu NPs inhibited Cd uptake in plants, while CuO NPs exhibited a combined effect. NPs caused damage to cells, and both the content of Cu and of Cd in plant subcellular components increased. The combined effect of Cd and copper-based nanoparticles can change the absorption of mineral nutrients by *Brassica* and reduce the nutritional value of crops.

This experiment explored the toxic effects of copper-based nanoparticles on the physiological and biochemical aspects of *Brassica* under Cd stress. However, in this experiment, the experimental method of hydroponics and a lower concentration of NPs were selected. Due to the complex toxicity mechanism of NPs, there may be some differences in the toxic effects caused by the soil environment and the increase in NPs concentration, which warrants further research to prove. The effects of the combination of Cd and NPs on cell and gene damage in plants may be a future research direction.

## Figures and Tables

**Table 1:** Effect of Cu NPs, CuO NPs and Cd on photosynthetic rate of *Brassica* from 1 to 7 days. The means are averaged from three replicates, and the error bars correspond to the standard deviations of the three values. Different letters above each column indicate a significant difference among treatments in the same group (*p* < 0.05).

	D1	D2	D3	D4	D5	D6	D7
Cd	39.68 ± 1.83 b	38.79 ± 0.65 c	38.80 ± 3.22 c	38.76 ± 3.16 b	37.91 ± 1.48 c	38.59 ± 1.82 c	38.46 ± 3.23 c
Cu NPs	41.37 ± 4.19 b	43.76 ± 2.11 ab	44.26 ± 1.61 ab	44.53 ± 4.84 ab	44.15 ± 2.74 b	44.53 ± 1.28 b	44.60 ± 2.28 ab
CuO NPs	47.30 ± 2.74 a	47.06 ± 3.13 a	47.57 ± 1.42 a	48.14 ± 5.41 a	48.42 ± 1.67 a	49.31 ± 2.61 a	49.86 ± 3.77 a
Cu NPs + Cd	40.87 ± 1.14 b	41.23 ± 1.22 bc	41.54 ± 1.40 bc	42.44 ± 1.88 ab	42.54 ± 0.70 b	42.90 ± 1.80 b	42.76 ± 1.47 bc
CuO NPs + Cd	43.96 ± 3.51 ab	43.48 ± 1.24 ab	43.44 ± 2.56 ab	43.51 ± 2.04 ab	43.42 ± 1.53 b	43.45 ± 2.25 b	45.98 ± 3.34 ab

**Table 2:** Effects of Cu NPs, CuO NPs and Cd on nutrient content. The mean is the mean of three replicates and the error bars correspond to the standard deviation of the three values. Different letters above each column indicate significant differences among treatments in the same group (*p* < 0.05).

Nutrition Elements		Cd	Cu NPs	CuO NPs	Cu NPs + Cd	CuO NPs + Cd
Zn	Leaves	90.54 ± 20.71 a	79.28 ± 14.20 ab	71.15 ± 7.23 ab	62.87 ± 6.01 b	69.45 ± 6.52 ab
	Roots	80.30 ± 11.96 b	82.60 ± 3.43 b	98.59 ± 8.42 ab	91.28 ± 8.32 ab	121.82 ± 37.70 a
Ca	Leaves	82.83 ± 0.45 a	74.54 ± 1.86 b	73.57 ± 4.32 b	63.90 ± 4.52 c	68.35 ± 6.98 bc
	Roots	77.28 ± 5.16 b	87.23 ± 2.53 ab	90.09 ± 4.50 a	91.03 ± 8.78 a	89.30 ± 5.32 a
Fe	Leaves	5.77 ± 0.03 a	2.76 ± 0.21 c	2.99 ± 0.18 bc	3.08 ± 0.07 bc	3.27 ± 0.22 b
	Roots	98.04 ± 7.98 b	99.66 ± 5.10 b	147.11 ± 28.01 a	130.31 ± 19.82 ab	114.49 ± 18.89 ab
Mg	Leaves	108.24 ± 4.42 a	88.25 ± 3.10 b	84.02 ± 3.23 b	83.87 ± 1.62 b	110.48 ± 3.55 a
	Roots	55.16 ± 3.31 c	53.72 ± 2.17 c	85.38 ± 10.34 a	80.46 ± 8.76 ab	72.38 ± 1.34 b
K	Leaves	945.79 ± 51.23 ab	911.33 ± 42.53 b	949.09 ± 26.97 ab	745.87 ± 22.82 c	1001.68 ± 46.02 a
	Roots	474.67 ± 43.25 c	493.99 ± 10.67 c	774.41 ± 79.53 a	602.24 ± 67.05 b	697.33 ± 43.84 ab
Mn	Leaves	118.64 ± 1.80 b	116.79 ± 7.25 b	97.33 ± 4.48 d	109.15 ± 2.02 c	147.43 ± 2.11 a
	Roots	40.12 ± 4.71 cd	34.86 ± 0.79 d	64.93 ± 14.16 a	49.12 ± 5.01 bc	54.35 ± 4.94 ab
Na	Leaves	21.22 ± 0.80 b	21.45 ± 0.91 b	18.07 ± 0.56 c	22.13 ± 0.28 b	27.25 ± 1.06 a
	Roots	93.52 ± 6.34 b	101.82 ± 5.99 ab	103.50 ± 12.14 ab	92.38 ± 7.96 b	108.56 ± 1.90 a

## Data Availability

Not applicable.

## Supplementary Materials

The following supporting information can be downloaded at: https://www.mdpi.com/article/10.3390/nano12091497/s1. Table S1: The composition and concentration of Hoagland nutrient solution.
