# Supplementary material for: Phytotoxicity and Accumulation of Copper-Based Nanoparticles in Brassica under Cadmium Stress
Source: Nanomaterials (Basel). 2022 Apr 28;12(9):1497. doi: 10.3390/nano12091497 (PMC9104374; doi:10.3390/nano12091497)
Supplement: Supplementary file 1 [file nanomaterials-12-01497-s001.zip › nanomaterials-1678100-supplementary.pdf]

Supplementary Materials

Phytotoxicity and Accumulation of Copper-Based Nanoparticles in *Brassica* under Cadmium Stress

Shiqi Wang [1,2,3], Yutong Fu [1,2,3], Shunan Zheng [4], Yingming Xu [2,3] and Yuebing Sun [2,3,*]

1 College of Resources and Environment, Northeast Agricultural University, Harbin 150030, China; wwp875822270@163.com (S.W.); 18845141255@163.com (Y.F.)
2 Key Laboratory of Original Agro-Environmental Pollution Prevention and Control, Ministry of Agriculture and Rural Affairs (MARA), Agro-Environmental Protection Institute, Tianjin 300191, China; ymxu1999@126.com
3 Tianjin Key Laboratory of Agro-Environment and Agro-Product Safety, Agro-Environmental Protection Institute, MARA, Tianjin 300191, China;
4 Rural Energy & Environment Agency, MARA, Beijing 100125, China; zhengshunan1234@163.com
* Correspondence: sunyuebing2008@126.com

Table S1. The composition and concentration of Hoagland nutrient solution

TK	Composition	Concentration(mg/L)
TK1	K_2SO_4	130.7
	KCl	7.5
	KH_2PO_4	34.0
TK2	$MgSO_4{\cdot}H_2O$	160.2
	$Ca(NO_3)_2{\cdot}4H_2O$	472.3
TK3	H_3BO_3	0.6
TK4	$CuSO_4{\cdot}5H_2O$	2.5×10^{-2}
	$ZnSO_4{\cdot}7H_2O$	0.3
	$MnSO_4{\cdot}H_2O$	0.2
	$(NH_4)_6Mo_7O_{24}{\cdot}4H_2O$	6.2×10^{-3}
TK5	$FeSO_4{\cdot}7H_2O$	27.8
	$Na_2EDTA{\cdot}2H_2O$	37.2
